# Artificial Dilatation of the Os Uteri

**Published:** 1853

**Authors:** T. E. Rawson


*Artificial Dilatation of the Os Uteri.*—By Dr. T. E. Rawson.

[Although Dr. Rawson is no advocate for meddlesome interference in cases of labor, he gives the following example of the value of artificial dilatation of the os uteri:]

A few years ago, I was sent for on the Tuesday morning to attend Mrs. B., living about six miles from my home. She was a rather stout person, of dark complexion, had been married about twelve months, and was fifty years of age. She had, moreover, lost one leg, and this was her first child. On my arrival, I was informed she had been suffering severe and regular pains for several hours. On making an examination, I found the pelvis well formed and roomy, but the os uteri was rigid and firmly closed. Her pains, which were strong, recurred about every seven or ten minutes. After staying several hours, without observing any relaxation of the os uteri, I left her, but was summoned to her again the same night. I found matters precisely in the same state, but she had become impatient and dispirited. I bled her in the arm in a full stream to syncope, hoping by this means to induce relaxation of the os uteri, but without effect. Her bowels were relieved by castor oil, and the next morning I gave her a full dose of opium, and left her. In the evening of Wednesday I visited her again. The pains had not diminished in force or frequency, but the os uteri had not as yet given way in the least. She had, however, become much more hopeful and cheerful, as I had before assured her there was no danger, and that it was a mere question of time and patience. She had had short intervals of sleep between the pains, and her appetite had much improved. I now gave her repeated doses of tartarized antimony, keeping up a constant nausea, but still without any relaxing effect on the os uteri. On the Thursday morning I had her placed in a warm bath, but to no purpose. I therefore ordered her to continue the nauseating doses of antimony, and again left her till the evening. I then found her still in the same general condition, hopeful, and without any symptoms of exhaustion, The pains were still strong and regular, no change in the os uteri.

What was to be done? I resolved on trying the effect of artificial dilatation. After some time, and with considerable difficulty, I succeeded in introducing the point of the index finger through the os uteri, then two fingers, and subsequently all the fingers and thumb conically disposed. By patient perseverance I gradually dilated the os uteri to the size of a crown piece; I then left her for the night, and on Friday morning found the membranes and head slightly protruding through the os. I then ruptured the membranes and gradually increased the dilatation, slipping the os uteri back to the broadest part of the head, No further progress was made dnring Friday, though the pains continued unabated in force and frequency. During Saturday, I gave her frequently repeated doses of ergot of rye, which had some effect in increasing the expulsive efforts of the uterus, but by Sunday the head of the fœtus had only reached the brim of the pelvis. After some little further progress had been made in the second stage, I applied the long forceps and slowly removed a large, healthy, and living child, on Monday morning. The mother made a rapid recovery, after one of the most tedious labors on record, having lasted about 150 hours!

I think it will be admitted that in this case artificial dilatation of the os uteri was not only justifiable, but was the only alternative under the cilcumstances. This instructive case proves how little mere tediousness constitutes an element of danger in labor. Probably the bleeding and the other antiphlogistic means prevented any febrile or inflammatory action. This case also exemplifies the great powers of enduranec of nature, when there are no special mechanical impediments. The pains never diminished in force from the beginning; the pulse retained its fulness, and the system its general powers to the last, except during the action of the antimony. She was more cheerful and hopeful, and enjoyed a better appetite on the last day than on the first.

I have met some few cases where the os uteri, only partially dilated, has been carried down even through the os externum; in such instances there can be no doubt of the propriety of artificial dilatation.

*Lancet*, July 23d. 1853, p. 86.

